# Neuropsychological predictors of conversion from mild cognitive impairment to Alzheimer’s disease: a feature selection ensemble combining stability and predictability

**DOI:** 10.1186/s12911-018-0710-y

**Published:** 2018-12-19

**Authors:** Telma Pereira, Francisco L. Ferreira, Sandra Cardoso, Dina Silva, Alexandre de Mendonça, Manuela Guerreiro, Sara C. Madeira, for the Alzheimer’s Disease Neuroimaging Initiative

**Affiliations:** 1 LASIGE, Faculdade de Ciências, Universidade de Lisboa, Lisbon, Portugal; 2 Instituto Superior Técnico, Universidade de Lisboa, Lisbon, Portugal; 3 Laboratório de Neurociências, Instituto de Medicina Molecular, Faculdade de Medicina, Universidade de Lisboa, Lisbon, Portugal; 4 Cognitive Neuroscience Research Group, Department of Psychology and Educational Sciences and Centre for Biomedical Research (CBMR), University of Algarve, Faro, Portugal

**Keywords:** Feature selection, Ensemble learning, Mild cognitive impairment, Alzheimer’s disease, Prognostic prediction, Neuropsychological data, Time windows

## Abstract

**Background:**

Predicting progression from Mild Cognitive Impairment (MCI) to Alzheimer’s Disease (AD) is an utmost open issue in AD-related research. Neuropsychological assessment has proven to be useful in identifying MCI patients who are likely to convert to dementia. However, the large battery of neuropsychological tests (NPTs) performed in clinical practice and the limited number of training examples are challenge to machine learning when learning prognostic models. In this context, it is paramount to pursue approaches that effectively seek for reduced sets of relevant features. Subsets of NPTs from which prognostic models can be learnt should not only be good predictors, but also stable, promoting generalizable and explainable models.

**Methods:**

We propose a feature selection (FS) ensemble combining stability and predictability to choose the most relevant NPTs for prognostic prediction in AD. First, we combine the outcome of multiple (filter and embedded) FS methods. Then, we use a wrapper-based approach optimizing both stability and predictability to compute the number of selected features. We use two large prospective studies (ADNI and the Portuguese Cognitive Complaints Cohort, CCC) to evaluate the approach and assess the predictive value of a large number of NPTs.

**Results:**

The best subsets of features include approximately 30 and 20 (from the original 79 and 40) features, for ADNI and CCC data, respectively, yielding stability above 0.89 and 0.95, and AUC above 0.87 and 0.82. Most NPTs learnt using the proposed feature selection ensemble have been identified in the literature as strong predictors of conversion from MCI to AD.

**Conclusions:**

The FS ensemble approach was able to *1)* identify subsets of stable and relevant predictors from a consensus of multiple FS methods using baseline NPTs and *2)* learn reliable prognostic models of conversion from MCI to AD using these subsets of features. The machine learning models learnt from these features outperformed the models trained without FS and achieved competitive results when compared to commonly used FS algorithms. Furthermore, the selected features are derived from a consensus of methods thus being more robust, while releasing users from choosing the most appropriate FS method to be used in their classification task.

**Electronic supplementary material:**

The online version of this article (10.1186/s12911-018-0710-y) contains supplementary material, which is available to authorized users.

## Introduction

Alzheimer’s disease (AD) is a neurodegenerative disorder with devastating effects on patients and their families and the leading cause of dementia [1]. The first symptom is frequently, but not always, difficulty in remembering new information, but progressive cognitive and functional decline follows [1]. On advanced stages, patients become unable to complete basic daily life activities, such as dressing, eating, and personal care [1]. Unfortunately, no treatment is available to revert or attenuate disease progression. Nowadays, more than 30 million people suffer from AD worldwide and its prevalence is expected to triple by 2050 [2], mainly due to population ageing. Although dementia affects mostly older people, there is a growing awareness of cases starting before the age of 65 [3]. Being one of the costliest chronic diseases, these numbers represent not only a true global epidemic, but also a huge socio-economic burden to modern societies [4]. Mild Cognitive Impairment (MCI) is considered a transition stage between healthy aging and dementia. MCI patients have cognitive complaints not interfering significantly with daily life activities. These patients are more likely to develop AD [5]. In this context, reliably predicting conversion of MCI to AD can help physicians to take decisions concerning patients’ treatment, patients’ participation in cognitive rehabilitation programs, and patients’ selection for clinical trials with novel drugs.

The last decades witnessed a boost in the emergence of machine learning approaches applied to AD-related research, recognized as powerful techniques to improve diagnostic and prognostic [6–13]. However, when analyzing clinical data, machine learning faces the challenge of learning from data with a large number of features and a reduced number of learning examples. Data is high dimensional and often heterogeneous leading to the well-known curse-of-dimensionality problem [14]. In this context, feature selection (FS), a data preprocessing procedure, is broadly used for dimensionality reduction [15–18]. Feature selection identifies subsets of relevant features, preserving (and putatively enhancing) the discriminative capability of the original set of features [16]. On the one hand, FS removes irrelevant features diminishing noise from data. On the other hand, using a smaller number of features reduces model complexity and prevents overfitting, improving learning performance by promoting generalization [16].

Feature selection algorithms may be categorized into three main classes: filter, wrapper, and embedded methods [15, 17]. The main difference between them relies on whether or not a learning algorithm is included in the selection process. Filter methods evaluate feature worth based on general characteristics of data (such as feature correlation) and are therefore independent of any learning algorithm. Wrapper methods, on the other hand, rely on the performance obtained by a given classifier to assess the importance of a subset of features. Wrapper methods accomplish better accuracy scores but are more prone to overfitting and computationally expensive for high dimensional datasets [17]. Filter methods are generally more efficient than wrapper methods although the emergent selected features may not be optimal to the target learning algorithm. In this context, embedded methods have been proposed as an in-between option among the aforementioned methods [19, 20]. Embedded methods join feature selection with model learning. As such, despite interacting with the learning algorithm, they are less computationally costly than wrapper methods, since no iterative evaluation of the subsets of features is done. The most widely used embedded methods are sparse learning based methods [17], where feature worth depends on the feature coefficients found to minimize errors while fitting the learning model. Alternatively to error minimization, stability selection [21] uses subsampling or bootstrapping to estimate the proper amount of regularization [8, 17] and find feature coefficients.

Regarding the output, feature selection methods can be classified as subset-based or ranking-based FS methods [15]. Subset-based FS returns a subset of the original feature cohort while the ranking-based FS returns the original feature cohort sorted by their worthiness (feature ranking).

When predicting conversion from MCI to AD, the subsets of selected features should fulfill three main requisites in order to be useful in clinical practice: 1) contain the most discriminative features independently of the FS algorithm used, 2) be robust to small data variations, and 3) be highly predictive of conversion from MCI to AD. Since there are several FS methods [17], and each has its own strengths and flaws, deciding which method is more suitable to the problem at hand is not trivial, requiring a deep understanding of both data and FS algorithms. In this scenario, we propose a feature selection ensemble combining stability and predictability (classification performance). To tackle 1) and 3) we propose the use of ensemble learning, an approach that combines the outcome of multiple learners (FS methods) trained to solve the same problem [18, 22–25]. Regarding 2) we propose to assess feature stability, here defined as the level of concordance between the subsets of features selected across the experiments [26].

Ensemble learning is based on the assumption that the output emergent from a consensus of learning algorithms outperforms that arising from a single method. In this work, we use heterogeneous ensembles in which the subsets of features selected by different FS methods (named base FS methods) are combined into a final subset of features [24]. This approach frees users from deciding the specific FS algorithm to use. Furthermore, FS results are less prone to be biased by the inherent characteristics of single FS algorithms and are thus putatively more generalizable. A robust subset of features is thus expected, as it results from a consensus between FS methods that rely on distinct search strategies. In this context, the higher the diversity among base FS methods the better [27].

Stability assesses the level of agreement between multiple subsets of features, emergent from different FS methods, experiments, or changes in data [26]. It is as important as predictability when users are not only interested in assessing the classification outcome but also on interpreting the selected features [26, 28, 29]. Despite the fact that much less attention has been devoted to the study of stability when compared to the emergence of FS algorithms, some stability measures have been proposed over the last years [26, 28, 30]. Kuncheva [28] introduced an index that measures stability by modelling the intersection between two subsets as a hypergeometric distribution. This index is widely used by FS researchers, mainly to compare the similarity between rankings of features derived by ensemble approaches [31, 32]. When compared to previously proposed indexes, based on the Jaccard index [33] or the relative Hamming distance [34], Kuncheva’s index has the advantage of having the property of correction for chance [28, 35]. It has, however, the limitation of requiring the sets of features to have the same cardinality. This issue shortens its application to ranking methods, where the number of features to keep is defined by the user (contrary to subset-based FS methods where the FS method controls the number of outputted features). Further comparison and description of stability measures can be found in [30, 35].

When seeking for reliable predictors of conversion from MCI to AD, thus improving prognostic models, powerful machine learning techniques have been increasingly used. In the scientific challenge promoted by Kaggle: “*A Machine learning neuroimaging challenge for automated diagnosis of Mild Cognitive Impairment*”, many competitive solutions benefited from complex feature selection approaches [10, 11, 13]. Nevertheless, despite the value of such FS approaches they have been mainly applied to neuroimaging and biochemical data [8–11, 36–39]. In contrast, studies using neuropsychological data, a standard way to characterize cognitive functioning in a clinical or research context, tend to rely on traditional statistical methods, such as regression-based methods (Discriminant Analysis, for instance) and survival regression models [40–42]. In this scenario, we believe it is fundamental to further explore the predictive power of neuropsychological tests (NPTs) using advanced machine learning techniques. NPTs are widely used in clinical practice in alternative to more expensive and often invasive approaches and achieved competitive results in predicting converting patients, when compared to biological biomarkers, such as brain imaging data (MRI and PET) and cerebrospinal fluid (CSF) [7, 9, 43–45]. Machine learning approaches have been shown to be more suitable to uncover hidden synergies between a large number of predictors than traditional statistical methods [46]. Furthermore, finding which NPTs are the most relevant for prognostic prediction would be helpful in clinical practice, enabling clinicians to reduce the number of tests that are performed, saving time, and potentially reducing the number of missing values in the NPTs data (occurring due to limitations of interview duration and patient fatigue), which may compromise the learning task.

In this paper, we propose a heterogeneous FS ensemble approach to automatically choose subsets of neuropsychological predictors of conversion from MCI to AD. The most relevant features are selected based on the combination of reduced sets of features learnt from multiple FS methods, preferentially with different theoretical foundations. We use ranking-based FS methods. Previous studies using heterogeneous ensembles [23, 24] differ from our proposal in what concerns the way multiple subsets of features are combined and optimized. In our study, the size of the subsets of features is found by combining their stability and classification performance. To our knowledge, it is the first time that stability and predictability are combined with this purpose in the context of FS in AD research, using ensemble learning.

We validated the proposed approach using two large datasets, the Alzheimer’s Disease Neuroimaging Initiative (ADNI) dataset [47] and the Cognitive Cohort Study (CCC) [48], and the selected neuropsychological predictors were compared in the task of predicting conversion from MCI to AD. However, we note that the proposed FS ensemble can be used to select relevant predictors in other diseases or prognostic problems.

## Methods

**Fig. 1:**
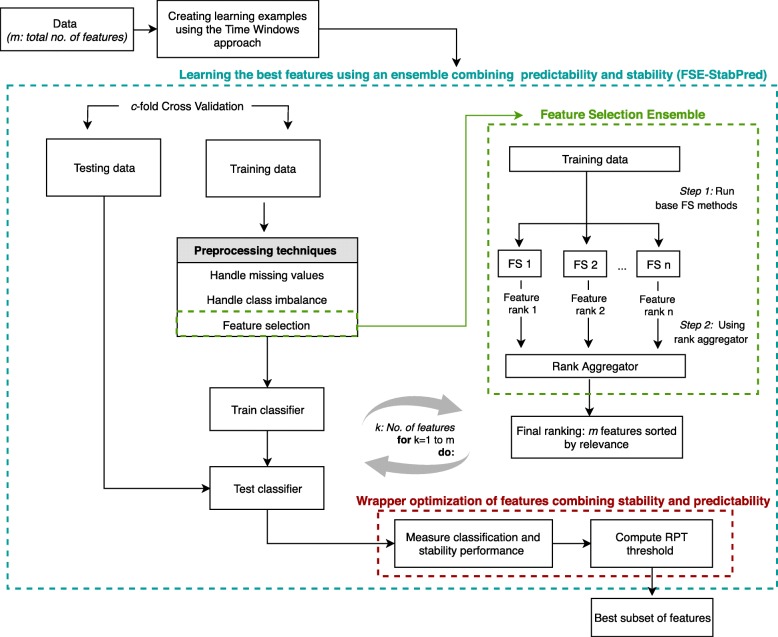
Workflow of the proposed feature selection ensemble approach combining stability and predictability (FSE-StabPred)

Figure 1 illustrates the proposed feature selection ensemble combining stability and predictability (abbreviated as *FSE-StabPred*), seeking for a robust, stable, and highly predictive set of neuropsychological features for prognostic prediction in MCI. This approach comprises two phases: *1)* finding a subset of features sorted by their relevance using ensemble learning and *2)* optimizing the subset of feature regarding its stability and predictability. The learning process follows a cross-validation (CV) procedure repeated with fold randomization to access model generalization.

In the first phase, training data (within each fold) is fed to an ensemble of ranking-based feature selection methods. Then, a rank aggregator is used to combine the rankings of features computed within the ensemble. A final ranking where features are sorted by their relevance is thus obtained.

In the second phase, a wrapper-based approach is used to optimize the size of the subset of features. Specifically, the classifier is run using an incremental subset of features including the top-ranked *k* features, where *k* ranges from 1 to the total number of features, *m*. The stability of these subsets of features, as well as the classification performance of the machine learning models (classifiers) learnt with these features are computed and averaged across CV-folds and fold randomization repetition. A threshold is then computed using these values of stability and classification performance to set the optimal size of the subset of features.

**Fig. 2:**
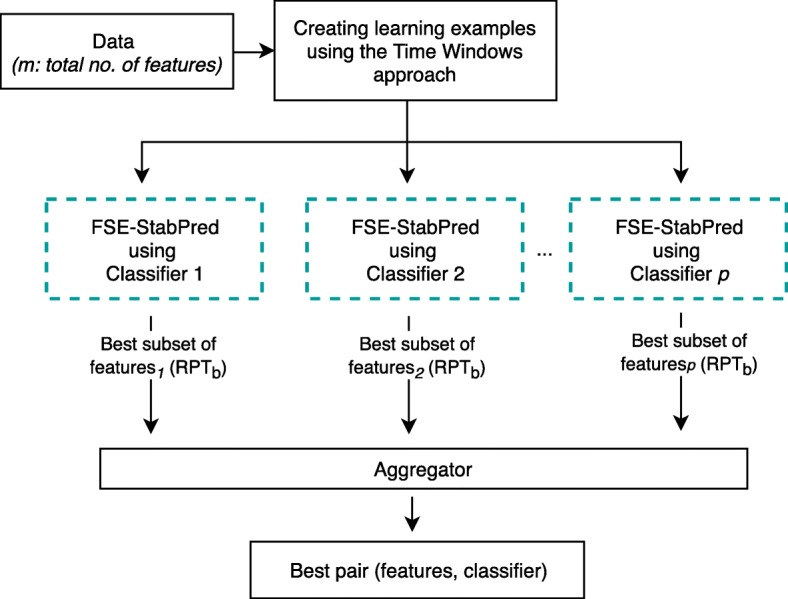
Workflow of the ensemble-based approach of the proposed FS ensemble combining stability and predictability (FSE-StabPred) using different classifiers

The optimal subset of features can vary with the classifier used to assess predictability. In this context, the proposed FS ensemble can be (optionally) run with multiple classifiers in an ensemble-based approach (Fig. 2). The emergent subsets of features are then combined in the aggregator, which selects the pair of features and classifier that yields the highest classification and/or stability performance. Such classifier is considered the most appropriate to learn the features of the data under study.

The proposed approach was tested in three clinically relevant time windows of conversion from MCI to AD. Differences and similarities between subsets of features (predictors) found for each time window (2, 3, and 4 years time windows) were studied.

Each step of the proposed FS ensemble approach is described in detail in the following subsections. We first describe data: ADNI and CCC. Then, we revise the procedure of creating learning examples using the Time Windows approach, first proposed in [12]. Follows a description of the main steps to learn the best features using an FS ensemble and a wrapper optimization of features combining stability and predictability. Finally, an ensemble-based approach to learn subsets of features with different classifiers is described.

### Data

Participants were selected from two large prospective studies: the ADNI project (http://adni.loni.usc.edu/) [47] and the Cognitive Complaints Cohort (CCC) [48]. Participants with clinical diagnosis of MCI at the baseline (first) assessment and who had at least one follow-up assessment were chosen. Demographic and neuropsychological data from different cognitive domains were selected in both datasets. Table A.1 shows the complete list of neuropsychological tests used in this work along with the respective mean average and missing values percentage for each dataset (ADNI and CCC) [See Additional file 1].

#### ADNI

ADNI was launched in 2003 as a public-private partnership led by Principal Investigator Michael W. Weiner [47]. Its goal is to find relevant biomarkers in all stages of AD to guide future clinical trials for new possible treatments. ADNI includes several biomarkers of Alzheimer’s disease beyond neuropsychological tests, such as cerebrospinal fluid, structural Magnetic Resonance Imaging (MRI), functional-MRI, Positron Emission Tomography, and other biological data. Data is collected from every ADNI participant at the baseline assessment, as well as annual follow-up consultations. Written informed consent was obtained from all participants and/or authorized representatives before protocol-specific procedures were carried out. This study was approved by ethical review boards in each participating institution. Participants were diagnosed with Mild Cognitive Impairment in the presence of a self-report (or via an informant) memory complaints without severe interference on daily live activities, objective memory deficit and absence of significant impairment on non-memory cognitive domains and of dementia. The NINCDS/ADRDA criteria were used to classify patients with probable AD.

In this work, we used 79 demographic and neuropsychological features from ADNI-2 patients (accessed in June 2017). NPTs include, but are not limited to, the Mini Mental State Examination (MMSE), the Alzheimer’s Disease Assessment Scale – cognitive subscale (ADAS-Cog), Clinical Dementia Rating (CDR) scale and Functional Assessment Questionnaire (FAQ). Table A.1 shows the complete list of features used in this study [See Additional file 1]. A total of 433 patients were analyzed: 122 MCI patients converted to dementia in a follow-up of 4 years while 311 preserved the MCI diagnostic for at least 2 years.

#### Cognitive complaints cohort

The Cognitive Complaints Cohort is a prospective study conducted at the Faculty of Medicine of Lisbon that recruits subjects with cognitive complaints, referred for a comprehensive neuropsychological assessment, with the aim of investigating their progression to dementia. The study was approved by the local ethics committee and all participants gave their written informed consent. The inclusion criteria for admission to CCC were the presence of cognitive complaints and undergoing a cognitive assessment with a neuropsychological battery designed to evaluate multiple cognitive domains and validated for the Portuguese population (*Bateria de Lisboa para Avaliação das Demências* – BLAD [49]). Participants were excluded from CCC if diagnosed with dementia (according to DSM-IV [50]) or other disorders that may cause cognitive impairment. Participants were diagnosed with Mild Cognitive Impairment when fulfilling the criteria of the MCI Working Group of the European Consortium on Alzheimer’s disease [51]. The MCI diagnosis could change to dementia, at follow-up, according to the DSM-IV [50] criteria. The dataset included 51 features covering demographic and neuropsychological data [See full list in Additional file 1]. A total of 584 patients were analysed: 175 MCI patients converted to dementia in a follow-up of 4 years while 409 preserved the MCI diagnostic for at least 2 years.

### Creating learning examples using time windows

Progression to dementia is characterized by a continuum cognitive, functional, and physical decline, which may last for decades [52]. MCI lies somewhere in this neurodegenerative process and thus passes throughout different stages of the disease. This leads to heterogeneous cohorts of MCI patients, which, if not considered, may affect the reliability of the prognostic models [12, 53–56]. In a previous work [12], we proposed to address the heterogeneity in MCI cohorts by creating time-homogenous groups regarding their time to conversion (named as Time Windows approach), when building the learning examples. This strategy was shown to improve the performance of the machine learning models to predict progression from MCI to dementia when compared to the models trained with the entire (heterogeneous) cohort of MCI patients. In this work, we followed the Time Windows strategy to create learning examples, which we briefly revise below.

For a given time window (2, 3 and 4 years in this study) we considered patients that converted to dementia within a predefined interval (using dementia diagnosis in one of the follow-up assessments up until the limit of the window). Those are labeled cMCI (converter MCI). On the other hand, patients that retained the MCI diagnosis up until the limit of the window or afterwards are included in the learning set labelled as sMCI (stable MCI). It is worth noting that the prognosis refers to a particular time window and might change if the considered time span changes. For instance, a given patient may be sMCI in a smaller window and a converting learning example in a wider window, using a posterior follow-up evaluation. Moreover, some cases might be disregarded if not enough follow-up evaluations are available, for a given time window. For instance, patients with the first follow-up assessment at 2.5 years from the baseline, and with dementia diagnosis, will create a learning example labelled as cMCI in the 3 (and 4) years time windows. However, no learning example is created for the 2-years time window. More details about the Time Windows approach may be found in [12].

Table 1 shows the proportion of learning examples for each time window of 2 to 4 years and datasets. These time windows were selected by pondering the follow-up length of both cohorts and the attempt to avoid skewed class proportion.

**Table 1:** Details on ADNI and CCC datasets for time windows of 2 to 4 years

	ADNI		CCC	
	sMCI	cMCI	sMCI	cMCI
2-Year window	311 (78%)	89 (22%)	409 (81%)	96 (19%)
3-Year window	235 (68%)	111 (32%)	310 (68%)	143 (32%)
4-Year window	143 (54%)	122 (46%)	227 (56%)	175 (44%)

Note: *sMCI* stable MCI, *cMCI* converter MCI. Class imbalance (per time window) is shown as % within brackets

Table 2 presents demographic characterization data. Differences among the cohorts of cMCI and sMCI patients were assessed by independent samples t-tests and *X*^2^ Person Chi-Square test to compare numerical and categorical measures, respectively. A *p*-value < 0.05 was assumed as statistically significant. Converting patients are older than those who remained MCI on both ADNI and CCC data. No statistical differences (*p* > 0.48) were found in formal education between converting and non-converting MCI patients from ADNI population. However, in CCC population, non-converting patients studied more years than those who converted. ADNI and CCC populations (sMCI and cMCI) are also statistically different regarding both age and education (*p* < 0.002, independent samples t-test). CCC included a more significant number of female participants while men were in the majority in ADNI. Moreover, ADNI participants were older and highly educated when compared to CCC participants.

**Table 2:** Baseline demographic characterization data

	Time window, years	ADNI			CCC		
		sMCI	cMCI	*p*-value	sMCI	cMCI	*p*-value
Age, years (M ± SD)	2	73.1 ± 7.8	74.4 ± 7.7	0.182	67.2 ± 8.9	72.5 ± 7.9	< 10^–7*^
	3	72.5 ± 7.6	74.9 ± 7.6	<0.006^*^	66.6 ± 8.8	72.3 ± 8.2	< 10^–10*^
	4	72.1 ± 7.3	74.8 ± 7.6	<0.004^*^	65.5 ± 9.1	71.9 ± 8.3	< 10^–12*^
Formal Education, years (M ± SD)	2	15.9 ± 2.7	16.2 ± 2.7	0.483	10.0 ± 4.7	8.9 ± 5.0	< 10^–7*^
	3	16.2 ± 2.7	16.0 ± 2.7	0.715	10.1 ± 4.8	8.6 ± 4.8	<0.003^*^
	4	16.1 ± 2.8	16.0 ± 2.6	0.895	10.4 ± 4.7	8.8 ± 4.8	<0.001^*^
Gender (male/female)	2	183/128	48/41	0.408	151/258	39/57	0.499
	3	136/99	64/47	0.969	119/191	50/93	0.483
	4	82/61	70/52	0.995	85/142	60/115	0.513

Group comparison (converter MCI vs stable MCI) were performed with Independent samples t-tests (Age and Formal Education) and *X*^2^ Person Chi-Square test (Gender). Statistically significant (*p* < 0.05) are marked with an asterisk (*)

Mean (M) and standard deviation (SD) values are illustrated

### Learning the best features using an ensemble combining predictability and stability

Once learning examples are created using the Time Windows approach, data is divided into *c* cross-validation folds (or subsets). Each time, one of the *c* subsets is used as testing data while the remaining *c-1* subsets are merged to form the training data. Each learning example is used exactly once in the testing data and *c-1* times in the training data. Data can be preprocessed to handle missing values, class imbalance, and dimensionality reduction. The latter is here performed using the proposed FS ensemble approach (Fig. 1, FSE-StabPred). First, a ranking of features is obtained from a consensus of different FS methods (Fig. 1, Feature Selection Ensemble). Then, the model is tuned to the training data using, at a time, the *k* top-selected features (where *k* ranges from 1 to the total number of features, *m*). Scores of stability and classification performance (evaluated on the testing data) are then used to compute a threshold reflecting the quality of each subset of features (Fig. 1, Wrapper optimization of features combining stability and predictability). The best subset of features is the one with the highest RPT threshold.

#### Feature selection ensemble

The FS ensemble used in this work is based on the heterogeneous ensemble approach proposed by Seijo-Pardo et al. [24]. Multiple rankings of features are created using different feature selection methods. These rankings of features are then combined into a final ranking of the most relevant features. By following this approach, we aim to obtain a robust subset of features as it results from the combination of methods that rely on distinct search strategies, that is, features found to be relevant independently of the technique used during the feature selection process. The FS ensemble has two steps: 1) running base feature selection methods and 2) using a ranking aggregator to combine multiple rankings of features.

##### Step 1: Using base feature selection methods to obtain multiple rankings

We used ranking-based feature selection algorithms with search criteria based on different metrics, as categorized in [17], to promote the ensemble diversity. Filter methods were preferred (over wrapper and embedded methods) since we target reduced sets of features independently of the learning algorithm applied afterwards. Moreover, we selected commonly used feature selection algorithms: ReliefF [57], Information Gain (MIM), Conditional Mutual Information Maximization (CMIM), Minimum Redundancy Maximum Relevance (MRMR), and Chi-Squared [58]. However, we also included two embedded methods used in related works [8, 22, 24]: the pruning-based method Recursive Feature Elimination using SVM (SVM-REF) [59] and the sparse learning-based method using the Logistic Loss (LL21) function via *l*_2, 1_ norm [60] regularizer. Wrapper methods were excluded due to their strong bias with the learning algorithm and high computational cost. The first five methods are univariate while the latter are multivariate methods. A further description of these FS methods may be found in [17].

##### Step 2: Using a rank aggregator to combine the multiple rankings

The multiple rankings computed by the different FS methods included in the ensemble must be combined into a single ranking using a combination method, named aggregator. Formally, an ensemble combining *n* different ranking-based FS methods produces a set *Q* = {*q*_*j*_, *j* = 1, …, *n*}, where *q*_*j*_ is either the ordered ranking of features (simple ranking) or the weighted ordered ranking of features (weighted ranking). Feature weights range from 0 to 1. The aggregator combines the weights (or the position in the ranking) of the features in the multiple rankings by using a relevance criteria. Arithmetic operations, such as mean, median, or maximum values are commonly used [24, 28, 31, 61, 62]. We used the mean aggregator which selects the average of the ranking position assigned by the FS methods. Then, features are sorted by their score into a final ranking. The higher the score, the more relevant the feature is.

#### Wrapper optimization of features combining stability and predictability

A final ranking of features is outputted from the FS ensemble module (Fig. 1). We assessed the quality of each subset of features, comprising the *k* top-ranked features, in terms of its stability and predictability (classification performance of the classifiers learnt with such features). These evaluation metrics are then used to compute a threshold, which reflects the worth of each subset of features. This threshold is optimized to find the best subset size and thus the most relevant features.

##### Measuring classification performance

Predictability is assessed by combining Area Under the ROC Curve (AUC) [63], sensitivity (proportion of actual converting patients (cMCI) who are correctly classified), and specificity (proportion of non-converting patients (sMCI) who are correctly identified). We used AUC since it is widely used in binary classification and is appropriate to deal with class imbalance. Specificity and sensitivity are frequently used in clinically-related research. We thus decided to combine the three evaluation metrics. Classification performance is given by:

1 $$ performance=\alpha\ AUC+\gamma \mathcal{F}\left( Sensitivity, Specificity\right) $$

where *α* and *γ*, with *α* + *γ* in [0, 1], are parameters that control the importance given to AUC or to the value given by $\mathcal{F}\left( Sensitivity, Specificity\right)$. $\mathcal{F}$ is a generic function to combine sensitivity and specificity scores, which may be tailored to the purpose of the learning task. In some application domains, it is critical to particularly avoid either false positives or false negatives. In this case, to evaluate the classification model we should focus on the specificity or sensitivity values, respectively, and thereby choose $\mathcal{F}$ that returns only the respective evaluation metric. AUC is kept in (1) with weight *α* to guarantee an acceptable overall classification performance, despite the bias introduced by the term *γ* that targets the specific evaluation metric (sensitivity or specificity). If, on the other side, we seek a supervised learning model as good as possible in discriminating both the positive and negative classes, we need to optimize both sensitivity and specificity. Different functions ($\mathcal{F}$) can be used to combine these metrics. We use simple arithmetic operations, such as mean or minimum, for the sake of interpretability. The minimum operator is suitable when one of the evaluation metrics will putatively perform worse than the other. This may occur in imbalanced data, for instance. When optimizing parameters or assessing the performance of models, the minimum allows targeting the worst performing evaluation metric, while guaranteeing that the other metric is at least equally good. In other words, we are optimizing an overall score (RPT or classification performance) while biasing this search to benefit the evaluation metric with the lowest scores, thus finding a good balance between sensitivity and specificity.

##### Measuring stability

Stability of feature selection may be understood as its sensitivity to small changes in data, experiments, or use of different methods [30]. We use Kuncheva’s index to assess the stability between rankings of features (3). The similarity (stability) between two sets of features is given by:

2 $$ Sim(k)=\frac{rm-{k}^2}{k\left(m-k\right)}, $$

where *r* represents common features between the two subsets of features, *k* is the subset size and *m* is the total number of features. The stability over the *n* subsets of features derived from the ensemble is given by the average similarity between those *n* pairs of features:

3 $$ Stability=\frac{2}{n\left(n-1\right)}{\sum}_{i=1}^{n-1}{\sum}_{j=i+1}^n\left({Sim}_i(k),{Sim}_j(k)\right). $$

This index is bounded by [−1, 1].

##### Computing the RPT threshold

There are different approaches to find the optimal subset size of a ranking of features: from keeping a percentage [24, 31, 62], computing the *log*2(*m*) or the Fisher’s discriminant ratio of the total number of features (*m*) [24, 62, 64], to strategies exploiting the classification error [28]. In this work, we use the robustness-performance trade-off (RPT), proposed in [22], to find the threshold that optimizes both the stability of the subset of features and its classification performance (predictability):

4 $$ RPT=\frac{\left({\beta}^2+1\right)\  stability\times predictability}{\beta^2 stability+ predictability} $$

where *β* is the parameter controlling the weight given to stability and classification performance.

### Computing the best subset of features with an ensemble-based approach using different classifiers

The search for the best subset of features relates to the classifier by means of the predictability in (4). In this context, we might (optionally) run the proposed FS ensemble with multiple classifiers and find the pair (features, classifier) that better fits the data under study (Fig. 2). In the first step, the FS ensemble approach combining predictability and stability (*FSE-StabPred*), described in Fig. 1, is run with *p* distinct classifiers. It returns, at least, *p* subsets of features (different parameters in (4) may be tested), which are then combined into a final one in the aggregator step. The aggregator selects the subset of features, computed using classifier *p*, that yields the maximum performance in a given evaluation metric (RPT, for instance). Other strategies can, however, be used to combine the best subsets of features found by each classifier *p*, such as outputting a final subset of features with the mean size of the *p* subsets of features.

### Classification settings

We used 10 × 5-fold stratified cross-validation and commonly used classifiers, relying on different learning approaches to the classification problem: Gaussian Naïve Bayes classifier (NB), Decision Tree (DT), Gaussian (SVM RBF) and Polynomial-kernel (SVM Poly) Support Vector Machines (SVMs), and Logistic Regression (LR). To deal with missing values, we removed features with a percentage of missing values above 20% and imputed the remaining using their mean or mode, in case they were numerical or nominal. This reduced to 40 (from 51) the number of features to be selected from CCC data while the original set of features from ADNI was maintained [See Additional file 1]. In addition, class imbalance was handled with Synthetic Minority Over-sampling Technique (SMOTE) [65]. SMOTE performs oversampling of the minority class with replacement. It creates synthetic instances by selecting (randomly) a set of instances from the minority class and perturbing the features by a random amount. SMOTE was only used when the class imbalance was superior to 70%. In order to ensure the validity of the results, all preprocessing techniques (FS, missing values imputation, and SMOTE) were applied to the training data within each cross-validation fold.

The feature selection ensemble was implemented using seven base FS methods: ReliefF, MIM, CMIM, MRMR, Chi-Squared, SVM-RFE, and LL21. Once learning the final rank of features (using the rank mean aggregator), the classifier was run (*m* times) using, at a time, the top-*k* ranked features (*k* ranging from 1 to the total number of features, *m*). This process was repeated for each round of 5 CV and each 10 iterations and thus, 50 (putatively) different aggregated rankings, and 50 × *m* models, were created. RPT values were then computed using the performance metrics achieved using these models. The best subset size is defined as the threshold that maximizes this threshold. Three *β* values were tested: *β* = 1 (equal weight to stability and classification performance), *β* = 0.1 (higher weight to stability) and *β* = 10 (higher weight to classification performance). Stability was measured using the index proposed by Kuncheva [28] while classification performance was assessed according to (1), where $\mathcal{F}\left( sensitivity, specificity\right)=\mathit{\min}\left( sensitivity, specificity\right)$ and *α* = *γ* = 0.5 (equal weight to both evaluation metrics). We used the minimum operator since we aim at finding the number of features that lead to a classification model as accurate as possible on classifying both converting and non-converting MCI patients, thus reaching a right balance between sensitivity and specificity. When running the FS ensemble approach combining stability and predictability using different classifiers, the aggregator outputs the pair of features and classifier with the highest RPT score.

Statistical significance of results was evaluated on the averaged classification performance given by (1) across 10×5-fold CV. Friedman Tests [66] were used to infer whether results obtained across different experiments, such as the base FS methods and the ensemble, or RPT thresholds with different *β* values, have statistically significant differences. Wilcoxon Signed Rank Tests were used for pairwise comparisons, with Bonferroni correction for multiple testing when needed. We used IBM SPSS Statistics 24 (released version 24.0.0.0) to run the statistical tests. The feature selection approach was implemented in Python using *scikit-learn* and the feature selection algorithms implemented in *scikit-feature* (http://featureselection.asu.edu) [17].

The proposed approach is applied to each dataset (ADNI and CCC) and time window (2, 3 and 4 years). We note, however, that the described feature selection approach may be used with any classifier, feature selection methods, and/or preprocessing options.

## Results

This section reports the outcome of the proposed FS ensemble when applied to ADNI and CCC data using neuropsychological data. We first analyse ensemble diversity concerning the base FS methods included. Follows an overall evaluation of results obtained with different classifiers. Then, we evaluate how stability and predictability vary with the number of top-selected features used to learn the prognostic model. Predictive performance of base FS methods is then compared with the FS ensemble approach. Finally, we discuss the clinical relevance of top-selected features for each dataset and time window.

### Diversity of FS methods used in the FS ensemble

Ensemble diversity is promoted by using seven algorithms based on different strategies to measure the worthiness of features. Since we believe that unstable base FS methods deteriorate the robustness of the ensemble, we analysed: 1) the stability of base FS methods individually, to decide whether they should or not join the ensemble, and 2) the pairwise stability between base FS methods to appraise ensemble diversity. Table 3 shows the stability score of each base FS method (in the diagonal) and the pairwise stability of base FS methods averaged over CV folds, repetitions, and the number of features (*k*) using ADNI data. Comparable results were obtained using CCC data [See Additional file 2] and are not included in the main text for the sake of readability.

**Table 3:** Individual and pairwise stability of the base FS algorithms used in the ensemble. Results are averaged over the 10 × 5 stratified CV and *m* subsets (for each possible subset size) for the 2-years (upper values), 3-years (middle values) and 4-years (bottom values), using ADNI data

	ReliefF	MIM	CMIM	MRMR	Chi-Squared	SVM-RFE	LL21
ReliefF	0.784 ± 0.127	–	–	–	–	–	–
	0.755 ± 0.147						
	0.728 ± 0.158						
MIM	0.601 ± 0.192	0.863 ± 0.112	–	–	–	–	–
	0.589 ± 0.215	0.862 ± 0.108					
	0.570 ± 0.208	0.861 ± 0.114					
CMIM	0.589 ± 0.243	0.704 ± 0.124	0.774 ± 0.152	–	–	–	–
	0.533 ± 0.242	0.667 ± 0.139	0.758 ± 0.164				
	0.479 ± 0.23	0.618 ± 0.168	0.739 ± 0.168				
MRMR	0.446 ± 0.211	0.301 ± 0.162	0.396 ± 0.174	0.858 ± 0.054	–	–	–
	0.371 ± 0.176	0.252 ± 0.144	0.379 ± 0.171	0.852 ± 0.064			
	0.367 ± 0.185	0.237 ± 0.161	0.390 ± 0.165	0.858 ± 0.054			
Chi-Squared	0.583 ± 0.1850	0.646 ± 0.118	0.529 ± 0.158	0.335 ± 0.212	0.871 ± 0.136	–	–
	0.591 ± 0.195	0.668 ± 0.130	0.514 ± 0.159	0.289 ± 0.193	0.874 ± 0.149		
	0.574 ± 0.217	0.668 ± 0.135	0.497 ± 0.167	0.286 ± 0.194	0.875 ± 0.148		
SVM-RFE	0.233 ± 0.097	0.184 ± 0.089	0.226 ± 0.107	0.323 ± 0.087	0.141 ± 0.068	0.302 ± 0.142	–
	0.219 ± 0.100	0.233 ± 0.089	0.251 ± 0.086	0.272 ± 0.064	0.184 ± 0.07	0.269 ± 0.076	
	0.217 ± 0.089	0.201 ± 0.089	0.227 ± 0.098	0.277 ± 0.059	0.193 ± 0.084	0.273 ± 0.097	
LL21	0.618 ± 0.224	0.617 ± 0.215	0.552 ± 0.236	0.414 ± 0.215	0.584 ± 0.202	0.200 ± 0.088	0.908 ± 0.056
	0.606 ± 0.22	0.613 ± 0.229	0.539 ± 0.241	0.353 ± 0.185	0.574 ± 0.203	0.206 ± 0.099	0.887 ± 0.064
	0.546 ± 0.253	0.565 ± 0.242	0.475 ± 0.235	0.324 ± 0.186	0.541 ± 0.209	0.184 ± 0.084	0.856 ± 0.087

SVM-RFE achieves a maximum individual stability score of 0.302 (Tables 2, 3-years time window) reflecting the inconsistency of the correspondent subsets of features. It underperforms the remaining FS methods, whose stability ranged between 0.728 to 0.908. We thus decided to exclude SVM-RFE from the ensemble. The highest stability is yielded by LL21, followed by Chi-squared, MIM, MRMR, ReliefF and CMIM methods.

The ensemble diversity is supported by the low scores of pairwise stability which range between 0.237 to 0.704. The features selected by MRMR are the most inconsistent with the remaining FS methods while LL21 has globally the highest pairwise stability.

### Computing the best subset of features with an ensemble-based approach using different classifiers

Tables 4 and 5 report the results obtained with the proposed FS ensemble (*FSE-StabPred*) using different classifiers, for each time window, using ADNI and CCC data, respectively. We recall that the best subset of features is that with the highest RPT, computed in the step “Wrapper optimization of features combining stability and predictability”. We tested three *β* values, when computing RPT, thus three putatively different subsets of features are found per classifier.

**Table 4:** Results obtained with the FS ensemble with subset size defined by RPT using *β* = 0.1 (upper values), *β* = 1 (middle values) and *β* = 10 (bottom values) for different classifiers. Results are averaged over 10 × 5 stratified cross validation, for each time window, using ADNI data

		AUC	Sensitivity	Specificity	Stability	# Features
2Y	NB	0.758 ± 0.00	0.599 ± 0.01	0.834 ± 0.00	1.0 ± 0.0	2
		0.839 ± 0.00	0.744 ± 0.01	0.779 ± 0.01	0.982 ± 0.01	17
		0.864 ± 0.00	0.791 ± 0.01	0.819 ± 0.01	0.912 ± 0.01	35
	SVM Poly	0.460 ± 0.08	0.323 ± 0.03	0.933 ± 0.00	1.0 ± 0.0	2
		0.849 ± 0.00	0.758 ± 0.01	0.797 ± 0.01	0.982 ± 0.01	17
		0.889 ± 0.01	0.789 ± 0.02	0.838 ± 0.01	0.913 ± 0.02	30
	SVM RBF	0.770 ± 0.00	0.571 ± 0.02	0.829 ± 0.01	1.0 ± 0.0	2
		0.864 ± 0.01	0.758 ± 0.02	0.825 ± 0.01	0.978 ± 0.02	22
		0.891 ± 0.01	0.777 ± 0.02	0.841 ± 0.01	0.913 ± 0.02	30
	DT	0.588 ± 0.02	0.446 ± 0.03	0.732 ± 0.03	1.0 ± 0.0	2
		0.706 ± 0.02	0.574 ± 0.04	0.839 ± 0.01	0.934 ± 0.02	38
		0.715 ± 0.03	0.568 ± 0.06	0.861 ± 0.01	0.919 ± 0.02	34
	LR	0.769 ± 0.00	0.637 ± 0.01	0.784 ± 0.00	1.0 ± 0.0	2
		0.846 ± 0.01	0.732 ± 0.03	0.821 ± 0.01	0.978 ± 0.02	22
		0.882 ± 0.01	0.727 ± 0.02	0.848 ± 0.01	0.913 ± 0.02	32
3Y	NB	0.772 ± 0.01	0.521 ± 0.01	0.901 ± 0.00	1.0 ± 0.0	2
		0.859 ± 0.00	0.761 ± 0.01	0.804 ± 0.01	0.985 ± 0.01	22
		0.872 ± 0.00	0.775 ± 0.02	0.829 ± 0.01	0.889 ± 0.01	30
	SVM Poly	0.734 ± 0.01	0.0 ± 0.0	1.0 ± 0.0	1.0 ± 0.0	2
		0.879 ± 0.00	0.584 ± 0.02	0.925 ± 0.01	0.927 ± 0.01	37
		0.876 ± 0.01	0.626 ± 0.02	0.912 ± 0.01	0.780 ± 0.02	55
	SVM RBF	0.777 ± 0.01	0.169 ± 0.02	0.982 ± 0.01	1.0 ± 0.0	2
		0.871 ± 0.01	0.614 ± 0.01	0.924 ± 0.01	0.985 ± 0.02	22
		0.872 ± 0.01	0.619 ± 0.02	0.914 ± 0.01	0.942 ± 0.01	25
	DT	0.602 ± 0.02	0.463 ± 0.02	0.742 ± 0.02	1.0 ± 0.0	2
		0.704 ± 0.02	0.603 ± 0.03	0.804 ± 0.02	0.959 ± 0.03	22
		0.719 ± 0.02	0.622 ± 0.03	0.816 ± 0.01	0.890 ± 0.02	33
	LR	0.777 ± 0.01	0.505 ± 0.01	0.920 ± 0.00	1.0 ± 0.0	2
		0.864 ± 0.01	0.658 ± 0.02	0.889 ± 0.01	0.985 ± 0.02	22
		0.864 ± 0.01	0.658 ± 0.02	0.889 ± 0.01	0.985 ± 0.02	22
4Y	NB	0.858 ± 0.03	0.779 ± 0.01	0.820 ± 0.01	0.937 ± 0.02	15
		0.891 ± 0.01	0.775 ± 0.01	0.844 ± 0.02	0.925 ± 0.02	36
		0.886 ± 0.00	0.789 ± 0.01	0.819 ± 0.01	0.895 ± 0.02	32
	SVM Poly	0.849 ± 0.01	0.706 ± 0.02	0.824 ± 0.02	0.937 ± 0.02	15
		0.904 ± 0.00	0.757 ± 0.02	0.884 ± 0.01	0.846 ± 0.02	36
		0.908 ± 0.01	0.757 ± 0.02	0.884 ± 0.01	0.847 ± 0.02	45
	SVM RBF	0.871 ± 0.01	0.702 ± 0.01	0.887 ± 0.01	0.937 ± 0.02	15
		0.901 ± 0.00	0.754 ± 0.02	0.863 ± 0.02	0.925 ± 0.02	10
		0.905 ± 0.01	0.758 ± 0.01	0.873 ± 0.03	0.829 ± 0.03	70
	DT	0.735 ± 0.03	0.708 ± 0.05	0.761 ± 0.02	0.937 ± 0.02	15
		0.735 ± 0.03	0.708 ± 0.05	0.761 ± 0.02	0.937 ± 0.02	15
		0.735 ± 0.03	0.708 ± 0.05	0.761 ± 0.02	0.937 ± 0.02	17
	LR	0.870 ± 0.01	0.745 ± 0.02	0.862 ± 0.01	0.937 ± 0.02	15
		0.870 ± 0.01	0.745 ± 0.02	0.862 ± 0.01	0.937 ± 0.02	15
		0.869 ± 0.01	0.751 ± 0.02	0.846 ± 0.01	0.905 ± 0.02	25

**Table 5:** Results obtained with the FS ensemble with subset size defined by RPT using *β* = 0.1 (upper values), *β* = 1 (middle values) and *β* = 10 (bottom values) for different classifiers. Results are averaged over 10 × 5 stratified cross validation, for each time window, using CCC data

		AUC	Sensitivity	Specificity	Stability	# Features
2Y	NB	0.803 ± 0.01	0.746 ± 0.01	0.681 ± 0.00	1.0 ± 0.0	9
		0.829 ± 0.00	0.771 ± 0.01	0.733 ± 0.01	0.971 ± 0.03	18
		0.829 ± 0.00	0.765 ± 0.01	0.744 ± 0.01	0.936 ± 0.01	20
	SVM Poly	0.815 ± 0.01	0.863 ± 0.01	0.767 ± 0.01	1.0 ± 0.0	9
		0.839 ± 0.00	0.789 ± 0.01	0.767 ± 0.01	0.965 ± 0.03	19
		0.841 ± 0.00	0.788 ± 0.01	0.758 ± 0.01	0.936 ± 0.01	20
	SVM RBF	0.820 ± 0.00	0.803 ± 0.02	0.673 ± 0.01	1.0 ± 0.0	9
		0.841 ± 0.00	0.771 ± 0.01	0.765 ± 0.01	0.965 ± 0.03	19
		0.841 ± 0.00	0.771 ± 0.01	0.765 ± 0.01	0.965 ± 0.03	19
	DT	0.616 ± 0.02	0.445 ± 0.03	0.786 ± 0.01	1.0 ± 0.0	9
		0.643 ± 0.02	0.578 ± 0.05	0.633 ± 0.03	0.918 ± 0.02	1
		0.643 ± 0.02	0.578 ± 0.05	0.633 ± 0.03	0.918 ± 0.02	1
	LR	0.811 ± 0.01	0.752 ± 0.02	0.726 ± 0.01	1.0 ± 0.0	9
		0.811 ± 0.01	0.752 ± 0.02	0.726 ± 0.01	1.0 ± 0.0	9
		0.821 ± 0.01	0.765 ± 0.01	0.765 ± 0.01	0.936 ± 0.01	20
3Y	NB	0.833 ± 0.00	0.749 ± 0.01	0.735 ± 0.01	1.0 ± 0.0	9
		0.857 ± 0.00	0.779 ± 0.01	0.772 ± 0.01	0.966 ± 0.01	19
		0.859 ± 0.00	0.778 ± 0.01	0.781 ± 0.01	0.950 ± 0.01	20
	SVM Poly	0.844 ± 0.00	0.0 ± 0.0	1.0 ± 0.0	1.0 ± 0.0	9
		0.872 ± 0.00	0.633 ± 0.01	0.886 ± 0.00	0.966 ± 0.02	19
		0.873 ± 0.01	0.643 ± 0.01	0.874 ± 0.00	0.909 ± 0.02	25
	SVM RBF	0.842 ± 0.00	0.582 ± 0.01	0.870 ± 0.01	1.0 ± 0.0	9
		0.873 ± 0.00	0.608 ± 0.01	0.891 ± 0.00	0.966 ± 0.02	19
		0.874 ± 0.00	0.612 ± 0.01	0.895 ± 0.01	0.950 ± 0.02	20
	DT	0.664 ± 0.02	0.556 ± 0.03	0.773 ± 0.03	1.0 ± 0.0	9
		0.686 ± 0.02	0.587 ± 0.03	0.784 ± 0.02	0.986 ± 0.02	12
		0.686 ± 0.02	0.587 ± 0.03	0.784 ± 0.02	0.986 ± 0.02	12
	LR	0.838 ± 0.01	0.619 ± 0.02	0.859 ± 0.00	1.0 ± 0.0	9
		0.838 ± 0.01	0.619 ± 0.02	0.859 ± 0.01	1.0 ± 0.0	9
		0.853 ± 0.01	0.635 ± 0.01	0.859 ± 0.01	0.950 ± 0.02	20
4Y	NB	0.852 ± 0.00	0.796 ± 0.01	0.768 ± 0.01	1.0 ± 0.0	9
		0.852 ± 0.00	0.796 ± 0.01	0.768 ± 0.01	1.0 ± 0.0	9
		0.868 ± 0.00	0.793 ± 0.01	0.788 ± 0.01	0.955 ± 0.02	19
	SVM Poly	0.853 ± 0.00	0.821 ± 0.01	0.720 ± 0.00	1.0 ± 0.0	9
		0.872 ± 0.00	0.775 ± 0.01	0.821 ± 0.01	0.959 ± 0.2	20
		0.872 ± 0.00	0.775 ± 0.01	0.821 ± 0.01	0.959 ± 0.02	20
	SVM RBF	0.858 ± 0.00	0.754 ± 0.01	0.798 ± 0.00	1.0 ± 0.0	9
		0.858 ± 0.00	0.754 ± 0.01	0.798 ± 0.00	1.0 ± 0.0	9
		0.871 ± 0.00	0.763 ± 0.01	0.820 ± 0.01	0.949 ± 0.03	16
	DT	0.675 ± 0.01	0.641 ± 0.02	0.713 ± 0.01	1.0 ± 0.0	2
		0.675 ± 0.01	0.641 ± 0.02	0.713 ± 0.01	1.0 ± 0.0	2
		0.682 ± 0.02	0.655 ± 0.03	0.717 ± 0.01	0.937 ± 0.04	14
	LR	0.852 ± 0.00	0.737 ± 0.01	0.801 ± 0.01	1.0 ± 0.0	9
		0.852 ± 0.00	0.737 ± 0.01	0.801 ± 0.01	1.0 ± 0.0	9
		0.742 ± 0.01	0.742 ± 0.01	0.803 ± 0.01	0.929 ± 0.01	15

The number of selected features varies considerably with the classifier and *β* used. In this study, it tends to increase with *β*, which reflects an increase in the weight assigned to predictability when computing RPT. When *β* = 0.1, the few features outputted by the FS ensemble are not able to properly classify converting MCI patients, revealed by the low sensitivity scores, while specificity yields higher scores. SVMs are particularly affected by this low number of features, producing the lowest sensitivity scores. However, their performance improves as the size of the subset of features increases. When using a larger subset of features, SVMs, NB and LR have similar classification performances although NB achieves, in general, a better balance between sensitivity and specificity. We observe that DT, on the other side, is the weakest classifier as it attains the poorest results in terms of classification performance for all time windows and datasets.

In what concerns the trade-off between stability and predictability, Naïve Bayes yielded highest RPT scores for all time windows and datasets, excepting for the 2-years time windows using ADNI and CCC data, where LR was superior. Therefore, according to the aggregator of the ensemble-based approach using different classifiers (which selects the pair of features and classifier with the highest RPT), the results reported in the next subsections use NB, and LR, to find the best subsets of features for the 3 and 4-years time windows, and the 2-years time window, respectively.

### Wrapper optimization of features combining stability and predictability

**Fig. 3:**
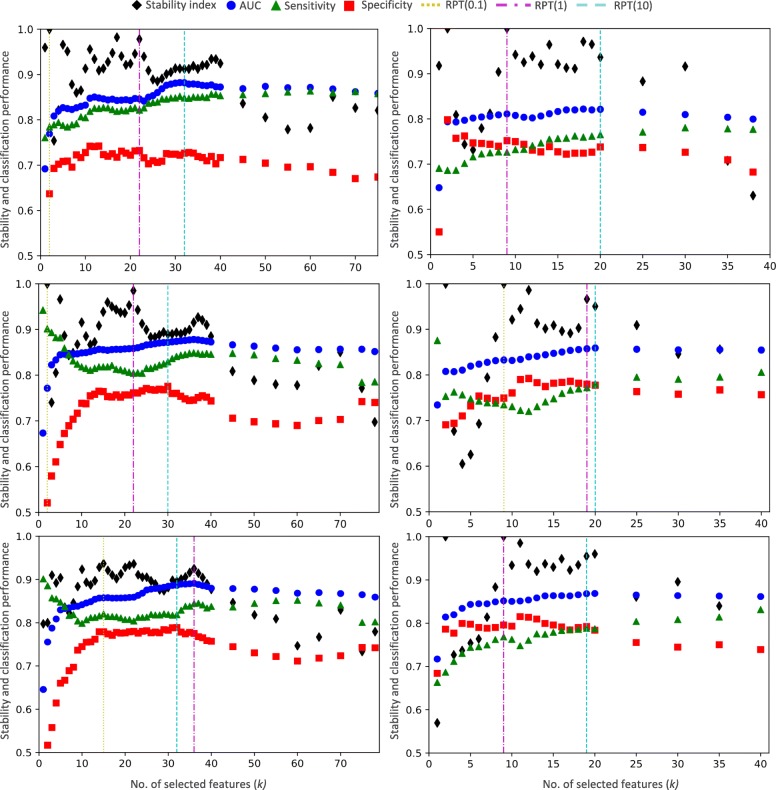
Stability and classification performance for subsets of features with different sizes (k) following 10 × 5 stratified CV and using time windows of 2-years (upper), 3-years (middle) and 4-years (bottom) obtained with ADNI (left panel) and CCC (right panel) data, using the NB and LR. RPT thresholds with β set as 0.1, 1 and 10 are illustrated

Figure 3 illustrates how feature stability and predictability vary with the size of the subset of features (*k*), for each time window in ADNI (*left panel*, Fig. 3) and CCC (*right panel*, Fig. 3) data, using NB (3 and 4-years time windows) and LR (2-years time window). Similar results were obtained for the remaining classifiers [See Additional file 3]. We note that features are ranked according to FS ensemble output.

Our results show that stability is more sensitive to the size of the subset of features than AUC (Fig. 3), corroborating previous studies [8, 38]. The stability curve is in general characterized by an initial fast growth with widely-dispersed values, having multiple local maximums, followed by a short plateau until it declines again. This large initial variance of stability values reflects the differences in the highest ranked features when using different FS methods and different data (CV folds). In this context, stability is an important factor to consider when optimizing the size of the subset of features to guarantee we choose (the number of) features that are consistently selected amongst cross-validation folds and feature selection methods.

The AUC curve, on the other hand, is characterized by a smooth and gradual growth until stabilization and, for larger subsets of features, by a slight decrease. Yet, sensitivity and specificity values show an accentuate variation with respect to the size of the subset of features (*k*). The small variation in AUC values is due to the synchronised increases and decreases in sensitivity and specificity (or vice-versa). In this context, we decided not to use AUC alone to assess predictability when computing RPT, since it would select thresholds for which either the sensitivity or specificity values were very low. Instead, we combined AUC with sensitivity and specificity (1) in order to pick thresholds with a good compromise between both evaluation metrics. This is in accordance with our aim of learning a model as accurate as possible in classifying both converting and non-converting patients.

RPT thresholds are strongly affected by the peaks of stability when *β* = 0.1 and *β* = 1 (Fig. 3). When *β* = 0.1, the respective thresholds match the first stability maximum, using ADNI data (*left panel*, Fig. 3). Due to the high stability scores, even when *β* = 1, stability plays the main role in determining the threshold value. Setting *β* = 10 mitigates this effect as the emergent cut-offs points (RPT thresholds) show a good trade-off between performances of stability and the remaining evaluation metrics. Statistical significant differences are found between the classification results obtained when learning the model with subsets of features delimited by each of the three RPT values (*β* = {0.1, 1, 10}) for all time windows and in both datasets (*p* < 0.0005, Friedman Test), except for the 2-years time window, using CCC data (*p* < 0.202, Friedman Test). In particular, *β* = 10 outperforms *β* = 0.1 (*p* < 0.005), across all time windows and datasets, and *β* = 1 (*p* < 0.005) in the 3 and 4-years time windows using ADNI and CCC data, respectively. No differences are found between *β* = 10 and *β* = 1 in the remaining datasets (*p* > 0.022). We thus considered RPT with *β* = 10 as the optimal threshold to ascertain the size of the subset of features. The best feature selection threshold finds subsets of size around 30 and 20 features for all time windows, using ADNI and CCC data, respectively.

### Comparing base feature selection methods with the ensemble

Tables 6 and 7 report the results obtained when using the proposed FS ensemble, each base FS method, and the original set of features to learn the prognostic model, in ADNI and CCC data, respectively. Classification results (computed according to (1)) are statistically significant for all time windows, as assessed by the Friedman Test [66] (*p* < 0.0005). Pairwise comparisons (using the Wilcoxon Signed Rank Test [66]) were then performed (with Bonferroni correction for multiple testing) to compare ensemble learning with the base FS methods and in the absence of feature selection. Results are significantly weaker when the entire set of features is used, instead of the subset given by the FS ensemble, over all time windows and using ADNI and CCC data (*p* < 0.005, Wilcoxon signed-rank tests). Apart from the superior classification results, using fewer features prevents overfitting, promotes generalization, and increases model interpretability. MRMR also lead to statistically worse classification results than the FS ensemble for the 3 and 4-years time windows, using ADNI and CCC datasets (*p* < 0.005). ReliefF outperforms the FS ensemble while Chi-Squared underperforms it in the 4-years time window (*p* < 0.005), using ADNI data. No statistically significant differences are found between the ensemble and the remaining methods MIM, CMIM, and LL21 (*p* > 0.009). Tackling redundancy between features while selecting them seems to be a strength for the problem under study as this is a common characteristic of MIM, CMIM, and LL21, which perform as good as the ensemble. Regarding CCC data, the ensemble approach outperforms Chi-Squared and LL21 for the 3-years time window (*p* < 0.007). No statistically significant differences are found in the remaining methods (*p* > 0.037) when using CCC data.

**Table 6:** Results obtained with the entire set of features, the FS ensemble and the individual FS algorithms for time-windows of a) 2-years, b) 3-years and c) 4-years, using ADNI data. Results are averaged over the 10 × 5 stratified cross validation with subset size defined by the optimized RPT threshold (*β* = 10)

		Ensemble	ReliefF	MIM	CMIM	MRMR	Chi-Squared	LL21	All features
2-years windows	AUC	0.882 ± 0.01	0.861 ± 0.01	0.865 ± 0.01	0.882 ± 0.01	0.859 ± 0.01	0.864 ± 0.0	0.851 ± 0.01	0.860 ± 0.01
	Sensitivity	0.727 ± 0.02	0.758 ± 0.02	0.754 ± 0.03	0.736 ± 0.03	0.749 ± 0.02	0.771 ± 0.01	0.752 ± 0.01	0.594 ± 0.02
	Specificity	0.848 ± 0.01	0.826 ± 0.01	0.833 ± 0.02	0.847 ± 0.01	0.803 ± 0.00	0.815 ± 0.01	0.821 ± 0.01	0.903 ± 0.01
	Stability	0.913 ± 0.02	0.888 ± 0.02	0.907 ± 0.02	0.892 ± 0.02	1.0 ± 0.0	1.0 ± 0.0	0.837 ± 0.02	–
	# Features	32	8	25	32	7	4	11	79
3-years windows	AUC	0.872 ± 0.004	0.857 ± 0.00	0.871 ± 0.00	0.872 ± 0.00	0.863 ± 0.00	0.859 ± 0.01	0.868 ± 0.00	0.835 ± 0.01
	Sensitivity	0.775 ± 0.018	0.784 ± 0.01	0.778 ± 0.01	0.782 ± 0.02	0.728 ± 0.01	0.776 ± 0.02	0.793 ± 0.02	0.714 ± 0.02
	Specificity	0.829 ± 0.011	0.817 ± 0.01	0.831 ± 0.01	0.827 ± 0.00	0.805 ± 0.01	0.841 ± 0.01	0.804 ± 0.01	0.782 ± 0.01
	Stability	0.889 ± 0.013	0.892 ± 0.02	0.913 ± 0.02	0.941 ± 0.01	0.789 ± 0.02	0.986 ± 0.02	0.944 ± 0.03	–
	# Features	30	15	23	29	70	10	45	79
4-years windows	AUC	0.886 ± 0.003	0.876 ± 0.00	0.881 ± 0.00	0.883 ± 0.01	0.867 ± 0.01	0.870 ± 0.01	0.872 ± 0.01	0.853 ± 0.006
	Sensitivity	0.789 ± 0.007	0.788 ± 0.01	0.799 ± 0.01	0.798 ± 0.01	0.732 ± 0.01	0.761 ± 0.01	0.792 ± 0.02	0.705 ± 0.009
	Specificity	0.819 ± 0.013	0.817 ± 0.02	0.818 ± 0.01	0.831 ± 0.01	0.815 ± 0.01	0.837 ± 0.01	0.819 ± 0.01	0.831 ± 0.017
	Stability	0.895 ± 0.023	0.839 ± 0.02	0.881 ± 0.02	0.899 ± 0.02	0.906 ± 0.05	0.93 ± 0.02	0.923 ± 0.03	–
	# Features	32	22	23	33	75	23	50	79

**Table 7:** Results obtained with the entire set of features, the FS ensemble and the individual FS algorithms for time-windows of a) 2-years, b) 3-years and c) 4-years, using CCC data. Results are averaged over the 10 × 5 stratified cross validation with subset size defined by the optimized RPT threshold (*β* = 10)

		Ensemble	ReliefF	MIM	CMIM	MRMR	Chi-Squared	LL21	All features
2-years windows	AUC	0.821 ± 0.00	0.813 ± 0.01	0.806 ± 0.01	0.817 ± 0.01	0.827 ± 0.01	0.820 ± 0.01	0.809 ± 0.01	0.814 ± 0.01
	Sensitivity	0.738 ± 0.02	0.744 ± 0.03	0.743 ± 0.02	0.735 ± 0.03	0.757 ± 0.02	0.742 ± 0.02	0.750 ± 0.02	0.385 ± 0.04
	Specificity	0.765 ± 0.01	0.758 ± 0.01	0.762 ± 0.01	0.767 ± 0.01	0.764 ± 0.01	0.763 ± 0.01	0.746 ± 0.01	0.920 ± 0.01
	Stability	0.936 ± 0.01	0.889 ± 0.03	0.928 ± 0.03	0.872 ± 0.03	0.975 ± 0.02	0.894 ± 0.02	0.986 ± 0.02	–
	# Features	20	14	16	25	16	20	12	40
3-years windows	AUC	0.859 ± 0.00	0.860 ± 0.00	0.861 ± 0.00	0.853 ± 0.04	0.855 ± 0.00	0.861 ± 0.00	0.863 ± 0.00	0.853 ± 0.00
	Sensitivity	0.778 ± 0.01	0.779 ± 0.01	0.778 ± 0.01	0.778 ± 0.01	0.762 ± 0.01	0.776 ± 0.01	0.775 ± 0.01	0.734 ± 0.01
	Specificity	0.781 ± 0.01	0.778 ± 0.01	0.784 ± 0.01	0.779 ± 0.01	0.785 ± 0.01	0.786 ± 0.00	0.792 ± 0.00	0.819 ± 0.00
	Stability	0.950 ± 0.02	0.922 ± 0.03	0.939 ± 0.01	0.885 ± 0.03	0.767 ± 0.03	0.992 ± 0.02	0.996 ± 0.02	–
	# Features	20	18	18	19	35	20	18	40
4-years windows	AUC	0.868 ± 0.00	0.868 ± 0.00	0.868 ± 0.00	0.865 ± 0.00	0.850 ± 0.00	0.869 ± 0.00	0.865 ± 0.00	0.859 ± 0.00
	Sensitivity	0.793 ± 0.01	0.773 ± 0.01	0.774 ± 0.01	0.795 ± 0.01	0.785 ± 0.01	0.793 ± 0.01	0.789 ± 0.01	0.729 ± 0.01
	Specificity	0.788 ± 0.00	0.792 ± 0.01	0.791 ± 0.00	0.789 ± 0.01	0.782 ± 0.01	0.789 ± 0.00	0.789 ± 0.00	0.841 ± 0.01
	Stability	0.955 ± 0.02	0.908 ± 0.02	0.951 ± 0.01	0.862 ± 0.3	0.802 ± 0.03	0.923 ± 0.02	0.909 ± 0.02	–
	# Features	19	18	16	18	12	16	15	40

In sum, although the FS ensemble approach does not outperform all base FS methods, it achieves competitive, or even superior, results in all experiments, being defeated only by ReliefF in the 4-years time window (ADNI).

### Features found as best prognostic predictors

Tables 8 and 9 show the top 30 and 20 ranked features found by the proposed FS ensemble (RPT threshold with *β* = 10), for each time window, using ADNI and CCC data, respectively. Features are sorted by relevance (ranking position, per time window, is indicated within brackets). Most features match across time windows on both datasets although with slight differences on the ranking positions.

**Table 8:** Top selected features using the ensemble approach with ADNI data (RPT threshold with *β set as* 10). Ranking positions of each feature are reported within brackets for the 2,3, and 4 years time windows, respectively

Common features across all time windows	
Trail Making Test (Part B) - time (1,1,1)	AVDELTOT: AVLT Recognition (14,15,20)
Forgetting Index (2,2,2)	Boston Test Naming (15,12,11)
AVTOT15: RAVLT 15 (3,3,4)	ADAS-Cog Q4: Delayed word recall (16,17,16)
ADAS-Cog Total 13 (4,4,5)	ADAS-Cog Q8: Word recognition (17,16,15)
Trail Making Test (Part A) - time (5,5,3)	MMSE (total) (18,20,17)
ADAS-Cog Total 11 (6,7,8)	ADAS-Cog Q1: Word recall (19,21,19)
AVTOT6: RAVLT 6 (7,6,7)	Letter Fluency (20,22,21)
Logical Memory Immediate (8,8,9)	Age (21,18,18)
Category Fluency (9,10,6)	Years of symptoms (22,19,22)
AVDEL30: RAVLT delay (10,9,10)	CDR: Orientation (25,26,26)
FAQ: Activities of Daily Living (11,13,13)	CDR: Home (26,23,23)
Logical Memory Delayed (12,11,12)	AVTOTB: AVLT Interference (27,25,25)
MOCADMDL (13,14,15)	
Common features across one or two time windows	
ADAS-Cog Q7: Orientation (23,24,-)	MMORIENTOT (29,-,-)
GDS (24,27,-)	ADAS-Cog Q13: Number cancelation (−,29,-)
Years of formal education (28,-,27)	CDR: Judgment and problem solving (−,-,29)
MMDLRECALL (−,28,24)	CDR: Community Affair (−,-,30)

**Table 9:** Top selected features using the ensemble approach with CCC data (RPT threshold with *β set as* 10). Ranking positions of each feature are reported within brackets for the 2,3, and 4 years time windows, respectively

Common features across all time windows	
Forgetting Index (1,1,1)	Verbal Paired-Associate Learning – Difficult (10,11,11)
Verbal Paired-Associate Learning – Total (2,2,2)	Verbal Paired-Associate Learning – Easy (11,10,10)
Cancelation Task – A’s time (3,4,6)	Word Recall (Total) (12,12,12)
Logical Memory Immediate A free recal (4,3,5)	Orientation (Total) (13,14,14)
Age (first symptoms) (5,8,7)	Raven Progressive Matrices (15,14,14)
Category Fluency (6,5,4)	Years of formal education (16,17,16)
Age (7,6,3)	Word Recall – Free recall (18,19,19)
Logical Memory A with Interference- free recall (8,7,9)	Cancelation Task – A’s total (19,18,18)
Logical Memory A Immediate Cued (9,9,8)	–
Common features across one or two time windows	
Interpretation of proverbs - (Verbal Abstraction) (17,-,20)	Calculation (19,-,-)
Information (−,16,17)	Orientation – Temporal (20,20,-)

A direct comparison between the most frequently selected neuropsychological measures in ADNI and CCC datasets is hard to establish mainly because they do not have a common neuropsychological battery. As an example, Trail Making Test (TMT) and Rey Auditory Verbal Learning Test (RAVLT) are amongst the highest ranked features in ADNI but could not be evaluated in CCC. While TMT was removed due to missing values constraints (preprocessing step), RAVLT does not make part of the CCC neuropsychological battery. Notwithstanding, most of the top selected NPTS in both datasets assess the same cognitive domains, supporting the concordance between the results. For instance, both TMT – Part A and Cancelation Task – A’s, top selected NPTs in ADNI and CCC, respectively, evaluate execution times. Moreover, tests to gauge memory impairment, such as Forgetting Index, Logical Memory (LM), RAVLT and Verbal Paired-Associate Learning (VPAL) are amongst the top selected NPTS in CCC and/or ADNI datasets. In addition, these NPTs have also been recognized as strong predictors of conversion from MCI to dementia due to Alzheimer’s Disease [8, 40].

## Discussion

The results demonstrate the effectiveness of the proposed feature selection ensemble combining stability and predictability (*FSE-StabPred*) to *1)* identify subsets of stable and relevant predictors from a consensus of multiple FS methods using baseline NPTs and *2)* learn reliable prognostic models of conversion from MCI to AD using these subsets of features. The prognostic models learnt from these features outperformed the models trained without FS and achieved competitive results when compared to commonly used FS algorithms and even superior sometimes. In fact, the FS ensemble was only beaten by ReliefF in the 4-years time window, using ADNI data (Tables 6 and 7). In this context, in our opinion, the ensemble approach should be preferred to individual feature selectors. On the one hand, it combines features coming from multiple methods with different search criteria thus being more robust. On the other hand, it releases users from deciding the most suitable FS method to use for a given task, without compromising results. In addition, by running the ensemble-based approach using different classifiers to find the best subset of features (Tables 4 and 5) we aim to guarantee that we choose the subset of features using the classifier better fitting the data under study. This classifier should then be used to learn the final prognostic model using the selected features. In this work, NB, and LR, were the best performing classifiers for the 3 and 4-years time windows, and the 2-years time window, respectively.

A recent comprehensive review on cognitive measures to predict conversion from MCI to AD [40] reports that individual neuropsychological tests show high specificity scores more often than high sensitivity scores when predicting progression from MCI to Alzheimer’s Disease. Our results corroborate this trend. In fact, for small subsets of features (less than 5 or 10 features for CCC and ADNI data, respectively) high specificity (and low sensitivity) scores are obtained in most time windows and datasets (Fig. 3). The large discrepancy between sensitivity and specificity attained with small subsets of features strengths the importance of our study: using sophisticated FS approaches and assessing a large number of neuropsychological measures together [46]. In fact, studying the predictive power of single (or small combinations of) NPTs [40] may not be sufficient to describe the complexity of this neurodegenerative process [46]. Moreover, as evidenced in Fig. 3, stability had a wide variation with the size of the subset of features used to learn the prognostic models, superior to AUC, corroborating previous studies [8, 38]. This stability’ variation reflects the differences in the highest ranked features when using different FS methods and different data (CV folds). With this in mind, stability is a key factor when optimizing the size of the subset of features to guarantee we choose (the number of) features that are constantly selected amongst cross-validation folds and features selection methods.

In what concerns prognostic prediction within time windows, results could not evidence a correlation between the choice of the most predictive subset of features and the time to conversion. The classification performance of the prognostic model improves throughout the time window growth, both with ADNI and CCC data (*Ensemble column*, Tables 6 and 7). This corroborates our previous findings [12] where prognostic models learnt with longer (4 and 5-years) time windows already achieved superior predictive performances. However, the behaviour of stability and classification performance curves, as well as the number of selected features, are similar across all time windows (Fig. 3). Furthermore, top-ranked features (Tables 8 and 9) are identical across the time windows.

Many of the highest ranked NPTs have been identified in the literature as being strong predictors of conversion from MCI to dementia due to Alzheimer’s Disease [8, 40]. Episodic memory (the ability to recall events that are specific to a time and place) has been seen as a hallmark risk feature for later development of AD [8, 67, 68]. It is usually the first domain to decline, with impairments being noticed up to 10 years before diagnosis [67, 69, 70], in population-based studies of preclinical AD. Episodic memory can be assessed using the Logical Memory (LM) test and through learning tasks evaluated in the RAVLT and Verbal Paired-Associate Learning (VPAL) test [40]. VPAL test has been effective in detecting MCI patients who will convert to dementia, particularly AD [67, 71]. In our study, sub-scores of the RAVLT and LM immediate, and three LM measures (immediate and delayed tasks) and VPAL test, are among the top-10 selected measures on ADNI and CCC, respectively. Moreover, forgetting index, a primarily test of memory used in [68], is the most relevant feature on CCC and the second most relevant feature on ADNI, for all time windows. This index evaluates the information successfully encoded, but lost in delayed recall and not recovered with the cued condition. Category verbal fluency has been identified as a strong predictor of conversion in our study (both in ADNI and CCC data), corroborating other researchers’ findings [45, 67]. Despite on different tests, execution times are considered discriminative of MCI patients who will (or not) convert to AD on ADNI (Trail Making Test - Part A) and CCC (Cancelation Task – A’s) data. Trail Making Test - time (Part A and B), ranked at least on the 5th position on ADNI dataset in our study, was also identified as a relevant predictor in [8]. Moreover, this test has been found to decline together with category fluency [67]. Our results support this finding, as both tests were on the top 10 features on both CCC and ADNI data.

Importantly, our feature selection ensemble approach proved to be valuable to identify predictors of MCI conversion do AD on two quite different datasets in two different countries. Furthermore, ADNI data has already been considered not representative of USA population, including mostly highly educated subjects [8, 72], and from this point of view, CCC is closer to the general Portuguese population. Besides demographic differences in these two cohorts, as well as the number and specific neuropsychological tests included, the proposed approach is able to identify equivalent (reflecting the same cognitive abilities known to be relevant for prediction of conversion to dementia in previous clinical studies) neuropsychological measures which are ranked top in both datasets.

Other researchers have applied machine learning strategies to automatically reduce the number of neuropsychological measures used in AD-related studies [6–8, 45]. Genetic algorithms have been used [7, 45] to choose subsets of relevant NPTs for prognostic prediction in AD. Our results are not only slightly superior regarding classification performance but select neuropsychological measures more in line with the literature [7] while using a larger patients’ cohort [45]. The combination of stability and feature selection was studied by Ye et al. [8]. The authors used sparse logistic regression with stability selection to find strong predictors from baseline ADNI measurements of demographic, genetic, cognitive and MRI data. Our feature selection ensemble approach (and classification model) achieved higher AUC values while using only neuropsychological data.

## Conclusions

Neuropsychological tests have proven their ability in discriminating between different stages of cognitive impairment [12, 40, 44]. However, the vast subjectivity and volume of the NPTs assessed in the clinical practice hampers the classification task. Feature selection is useful not only to automatically select the best NPTs to predict whether a MCI patient is likely (or not) to become demented in the future but also to improve model interpretability and classifier performance, which is often constraint by a small number of learning examples.

We proposed a heterogeneous ensemble approach to tackle feature selection where stability and predictability are combined to find the optimal subset of features. A subset of stable features is thus reached by choosing features selected from a consensus of different FS methods and keeping the top-selected features that optimize stability and predictability. Subsets of features may be (optionally) optimized by tuning the classifier used to assess predictability to the data under study. Results showed that the proposed FS ensemble is suitable to optimize the set of neuropsychological tests required to learn trustworthy prognostic models in AD. Although the ensemble approach did not outperform all base FS methods (run individually), its results were competitive in all experiments and even superior to base FS methods sometimes. As such, it is worth using the proposed FS ensemble approach as performance is not compromised, there is no need to choose the FS algorithm more suitable to the problem at hand, and the set of features result from a consensus of FS methods.

Our study has advantages over others [7, 8, 40] since it uses two large patients cohorts (one publicly available, ADNI, and a private Portuguese cohort, CCC) to validate the approach, evaluates the stability of the reduced subsets of features (its sensitivity to data perturbation), and considers a more significant number of baseline tests of cognitive functioning (total of 79 and 40 features from ADNI and CCC, respectively).

**ENDNOTES**

^a^ Data used in preparation of this article were obtained from the Alzheimer’s Disease Neuroimaging Initiative (ADNI) database (adni.loni.usc.edu). As such, the investigators within the ADNI contributed to the design and implementation of ADNI and/or provided data but did not participate in analysis or writing of this report. A complete listing of ADNI investigators can be found at: http://adni.loni.usc.edu/wp-content/uploads/how_to_apply/ADNI_Acknowledgement_List.pdf

## Additional files

Additional file 1: Description of the neuropsychological data of the CCC and ADNI sample (M ± SD: mean ± standard deviation and %MV: percentage or missing values are reported). (DOCX 45 kb)

Additional file 2: Individual and pairwise stability of the base FS algorithms used in the ensemble, using CCC data. (DOCX 61 kb)

Additional file 3: Stability and classification performance of classification models learnt with an incremental number of (ranked) features and using NB, DT, LR, SVM Poly and SVM RBF, per time windows, using ADNI and CCC data. RPT thresholds with β set as 0.1, 1 and 10 are illustrated. (DOCX 2920 kb)

## Abbreviations

AD: Alzheimer’s Disease
ADAS-Cog: Alzheimer’s Disease Assessment Scale – cognitive subscale
AUC: Area Under the ROC Curve
CCC: Cognitive Complaints Cohort
cMCI: converter MCI
CMIM: Conditional Mutual Information Maximization
CVLT: California Verbal Learning Test
FS: Feature selection
FSE-StabPred: Feature selection ensemble combining stability and predictability
LL21: Logistic Loss (LL21)
LM: Logical Memory Test
MCI: Mild Cognitive Impairment
MIM: Information Gain
MMSE: Mini-Mental State Examination
MRMR: Minimum Redundancy Maximum Relevance
NPTs: Neuropsychological tests
RAVLT: Rey Auditory Verbal learning Test
RPT: Robustness Performance trade-off
sMCI: stable MCI
SVM-REF: Recursive Feature Elimination
VPAL: Verbal Paired-Associate Learning
