# Supplementary material for: Neuropsychological predictors of conversion from mild cognitive impairment to Alzheimer’s disease: a feature selection ensemble combining stability and predictability
Source: BMC Med Inform Decis Mak. 2018 Dec 19;18:137. doi: 10.1186/s12911-018-0710-y (PMC6299964; doi:10.1186/s12911-018-0710-y)
Supplement: Supplementary file 1 — Description of the neuropsychological data of the CCC and ADNI sample (M ± SD: mean ± standard deviation and %MV: percentage or missing values are reported). (DOCX 45 kb) [file 12911_2018_710_MOESM1_ESM.docx]

**Table A.1.** Neuropsychological data of the CCC sample (M±SD: mean ± standard deviation and %MV: percentage or missing values are reported). Features considered as best predictors appears in bold.

|  | Neuropsychological test | sMCI | | cMCI | |
| --- | --- | --- | --- | --- | --- |
|  |  | **M**$\boldsymbol{\pm}$**SD** | **%MV** | **M**$\boldsymbol{\pm}$**SD** | **%MV** |
| BLAD  (Bateria de Lisboa para Avaliação das Demências) | *Cancelation Task – A’s cuts* | 15.33 ± 1.21 | 14% | 15.07 ± 1.51 | 10% |
|  | ***Cancelation Task- A’s time*** | 39.53 ± 16.51 | 15% | 47.49 ± 17.40 | 10% |
|  | ***Cancelation Task – A’s total*** | 4.47 ± 1.92 | 15% | 3.58 ± 1.13 | 10% |
|  | *Digit Span – Forward* | 5.00 ± 0.78 | 3% | 4.71 ± 0.68 | 1% |
|  | *Digit Span – Backward* | 3.83 ± 1.03 | 3% | 3.39 ± 0.95 | 1% |
|  | *Digit Span – Total* | 8.83 ± 1.49 | 3% | 8.12 ± 1.40 | 1% |
|  | ***Verbal Paired-Associate Learning – Easy*** | 14.15 ± 3.33 | 4% | 11.86± 3.71 | 3% |
|  | ***Verbal Paired-Associate Learning –Difficult*** | 4.39 ± 3.59 | 4% | 1.33 ± 2.06 | 3% |
|  | ***Verbal Paired-Associate Learning – Total*** | 11.45 ± 4.78 | 4% | 7.27 ± 3.28 | 3% |
|  | ***Logical Memory A Immediate free recall*** | 9.39 ± 4.43 | 3% | 5.49 ± 3.98 | 2% |
|  | ***Logical Memory A Immediate Cued*** | 12.21 ± 4.18 | 17% | 8.57 ± 4.28 | 10% |
|  | ***Logical Memory A with Interference- free recall*** | 8.50 ± 4.94 | 7% | 3.76 ± 3.98 | 10% |
|  | *Recovery from logical memory A immediate free recall* | 2.37 ± 1.10 | 18% | 2.81 ± 1.12 | 22% |
|  | ***^(1)^Forgetting Index*** | -9.47 ± 66.11 | 8% | -42.97 ± 52.07 | 14% |
|  | ***Word Recall – Free recall*** | 4.73 ± 3.42 | 14% | 2.25 ± 2.71 | 6% |
|  | *Word Recall – Cued* | 3.72 ± 1.99 | 14% | 4.18 ± 1.87 | 6% |
|  | *Word Recall – Recognition* | 1.13 ± 0.87 | 14% | 1.44 ± 0.94 | 6% |
|  | ***Word Recall (Total)*** | 9.59 ± 2.72 | 13% | 7.86 ± 2.26 | 6% |
|  | ***Information*** | 18.15 ± 2.61 | 23% | 16.42 ± 3.86 | 14% |
|  | ***Orientation (Total)*** | 14.26 ± 1.38 | 6% | 12.49 ± 2.39 | 5% |
|  | *Orientation – Personal* | 4.94 ± 0.36 | 6% | 4.76 ± 0.57 | 5% |
|  | *Orientation – Spatial* | 2.99 ± 0.18 | 6% | 2.91 ± 0.34 | 5% |
|  | ***Orientation – Temporal*** | 6.33 ± 1.18 | 6% | 4.84 ± 1.96 | 5% |
|  | ***Category Fluency*** | 16.56 ± 4.89 | 3% | 13.04 ± 4.30 | 2% |
|  | *Motor Initiative* | 2.76 ± .066 | 7% | 2.61 ± 0.76 | 3% |
|  | *Graphomotor Initiative* | 1.82± 0.39 | 11% | 1.69 ± 0.50 | 11% |
|  | *Clock Draw* | 2.73 ± 0.56 | 6% | 2.47 ± 0.73 | 5% |
|  | *Cube Draw* | 2.55 ± 0.75 | 19% | 2.16 ± 0.92 | 23% |
|  | ***Calculation*** | 12.73 ± 2.29 | 15% | 11.88 ± 2.99 | 14% |
|  | ***Interpretation of Proverbs –***  ***(Verbal Abstraction)*** | 7.42 ± 1.65 | 5% | 6.16 ± 1.80 | 3% |
|  | ***Raven Progressive Matrices*** | 9.09 ± 2.23 | 10% | 7.37 ± 2.56 | 10% |

***^(1)^*** *Forgetting Index = [(LM delayed recall – LM immediate)/LM immediate]*$\times$*100.*

**Table A.2.** Neuropsychological data of the ADNI sample (M±SD: mean ± standard deviation and %MV: percentage or missing values are reported). Features considered as best predictors appears in bold.

|  | Neuropsychological test | sMCI | | cMCI | |
| --- | --- | --- | --- | --- | --- |
|  |  | **M**$\boldsymbol{\pm}$**SD** | **%MV** | **M**$\boldsymbol{\pm}$**SD** | **%MV** |
| Alzheimer’s Disease Assessment Scale – cognitive subscale (ADAS-Cog) | ***ADAS-Cog Q1: Word recall*** | 3.87 ± 1.43 | 0% | 5.15 ± 1.51 | 0% |
|  | *ADAS-Cog Q2: Commands* | 0.96 ± 2.69 | 0% | 0.28 ± 0.67 | 0% |
|  | *ADAS-Cog Q3: Constructional Praxis* | 0.49 ± 0.55 | 0% | 0.56 ± 0.60 | 0% |
|  | ***ADAS-Cog Q4: Delayed word recall*** | 4.43 ± 2.35 | 0% | 6.88 ± 2.42 | 0% |
|  | *ADAS-Cog Q5: naming Objects and Fingers* | 0.11 ± 0.35 | 0% | 0.31 ± 0.63 | 0% |
|  | *ADAS-Cog Q6: Ideational Praxis* | 0.05 ± 0.22 | 0% | 0.12 ± 0.32 | 0% |
|  | ***ADAS-Cog Q7: Orientation*** | 0.26 ± 0.58 | 0% | 0.72 ± 1.06 | 0% |
|  | ***ADAS-Cog Q8: Word recognition*** | 3.16 ± 2.31 | 0% | 5.13 ± 3.02 | 0% |
|  | *ADAS-Cog Q9: Recall* | 0.01 ± 0.09 | 0% | 0.15 ± 0.61 | 0% |
|  | *ADAS-Cog Q10: Comprehension of Spoken Language* | 0.08 ± 3.26 | 0% | 0.12 ± 0.43 | 0% |
|  | *ADAS-Cog Q11: Word-finding Difficulty* | 0.27 ± 0.52 | 0% | 0.44 ± 0.75 | 0% |
|  | *ADAS-Cog Q12: Language* | 0.06 ± 0.29 | 0% | 0.01 ± 0.39 | 0% |
|  | ***ADAS-Cog Q13: Number cancelation*** | 0.53 ± 0.74 | 0% | 0.93 ± 1.08 | 0% |
|  | ***ADAS-Cog Total 11*** | 8.46 ± 3.62 | 0% | 13.06 ± 5.71 | 0% |
|  | ***ADAS-Cog Total 13*** | 13.40 ± 5.65 | 0% | 20.87 ± 7.58 | 0% |
| Clinical Dementia Rating Scale (CDR) | *CDR: Memory* | 0:12\|0.5:279\|1:17 | 1% | 0:2\|0.5:82\|1:38 | 0% |
|  | ***CDR: Orientation*** | 0:196\|0.5:109\|1:3 | 1% | 0:45\|0.5:57\|1:20 | 0% |
|  | ***CDR :Judgment and Problem Solving Score*** | 0:130\|0.5:173\|1:5 | 1% | 0:18\|0.5:92\|1:12 | 0% |
|  | ***CDR: Community*** | 0:244\|0.5:62\|1:2 | 1% | 0:68\|0.5:47\|1:7 | 0% |
|  | ***CDR: Home and hobbies*** | 0:224\|0.5:73\|1:11 | 1% | 0:45\|0.5:60\|1:16\|2:1 | 0% |
|  | *CDR: Personal care* | 0:302\|1:6 | 1% | 0:112\|1:9\|2:1 | 0% |
|  | *CDR (Total)* | 0:10\|0.5:298 | 1% | 0.5:116\|1:6 | 0% |
| Functional Assessment Questionnaire (FAQ) | ***FAQ: Activities of Daily Living*** | 2.23 ± 3.38 | 1% | 5.7 ± 0.44 | 2% |
| Geriatric Depression Scale | ***GDS*** | 1.77 ± 1.65 | 0% | 1.98 ± 1.64 | 0% |
| Mini Mental State Examination (MMSE) | *MMREPEAT* | 1:263\|2:48 | 0% | 1:102\|2:20 | 0% |
|  | *MMREAD* | 1:308\|2:3 | 0% | 1:120\|2:2 | 0% |
|  | *MMWRITE* | 1:305\|2:6 | 0% | 1:122 | 0% |
|  | *MMDRAW* | 1:286\|2:25 | 0% | 1:109\|2:13 | 0% |
|  | ***MMSE (Total)*** | 28.13 ± 1.71 | 0% | 27.06 ± 1.96 | 0% |
|  | *^(1)^MMORIENTTEMP* | 4.80 ± 0.47 | 0% | 4.51 ± 0.79 | 0% |
|  | *^(2)^MMORIENTSPAC* | 4.75 ± 0.48 | 0% | 4.69 ± 0.60 | 0% |
|  | *^(3)^MMIMRECALL* | 2.99 ± 0.08 | 0% | 2.98 ± 0.20 | 0% |
|  | *^(4)^MMATTENTION* | 4.70 ± 0.79 | 0% | 4.69 ± 0.86 | 0% |
|  | *^(5)^****MMDLRECALL*** | 2.25 ± 0.97 | 0% | 1.64 ± 1.12 | 0% |
|  | *^(6)^MMLANGUAGE* | 2:310\|2:1 | 0% | 1.98 ± 0.20 | 0% |
|  | *^(7)^MMLANGTOT* | 8.64 ± 0.59 | 0% | 8.56 ± 0.66 | 0% |
|  | *^(8)^****MMORIENTTOT*** | 9.55 ± 0.71 | 0% | 9.19 ± 1.0 | 0% |
|  | *^(9)^MMCOMMAND* | 2.90 ± 0.31 | 0% | 2.87 ± 0.34 | 0% |
| Montreal Cognitive Assessment (MoCA) | *TRAILS* | 0:43\|1:268 | 0% | 0:30\|1:92 | 0% |
|  | *cube draw* | 0:99\|1:212 | 0% | 0:56\|1:66 | 0% |
|  | *Digit Span – Forward* | 0:16\|1:295 | 0% | 0:8\|1:114 | 0% |
|  | *Digit Span – Backward* | 0:31\|1:280 | 0% | 0:17\|1:105 | 0% |
|  | *LETTERS* | 0.72 ± 2.01 | 0% | 0.79 ± 2.18 | 1% |
|  | ***Letter Fluency*** | 13.39 ± 4.65 | 0% | 13.17 ± 4.76 | 0% |
|  | *^(10)^MOCACLOCK* | 2.67 ± 0.55 | 0% | 2.45 ± 0.67 | 1% |
|  | *^(11)^MOCANAMING* | 2.88 ± 0.34 | 0% | 2.74 ± 0.49 | 1% |
|  | *^(12)^MOCAMIMT1* | 4.48 ± 0.80 | 0% | 4.19 ± 0.99 | 1% |
|  | *^(13)^MOCAMIMT2* | 4.76 ± 0.63 | 0% | 4.55 ± 0.79 | 1% |
|  | *^(14)^MOCASERIAL* | 4.26 ± 1.14 | 0% | 3.93 ± 1.33 | 1% |
|  | *^(15)^MOCAREPEAT* | 1.58 ± 0.64 | 0% | 1.51 ± 0.71 | 1% |
|  | *^(16)^MOCAABS* | 1.68 ± 0.59 | 0% | 1.56 ± 0.68 | 1% |
|  | *^(17)^****MOCAMDL*** | 8.05 ± 3.7 | 0% | 6.0 ± 3.37 | 1% |
|  | *^(18)^MOCAORIETTEMP* | 3.81 ± 0.46 | 0% | 3.05 ± 0.75 | 0% |
|  | *^(19)^MOCAORIETSPAC* | 1.94 ± 0.25 | 0% | 1.92 ± 0.28 | 0% |
| Neuropsychological Battery | ***Logical Memory Immediate*** | 10.68 ± 3.56 | 0% | 7.84 ± 4.39 | 0% |
|  | ***Logical Memory Delayed*** | 8.35 ± 3.82 | 0% | 4.86 ± 4.52 | 1% |
|  | *LDELCUE* | 0.12 ± 0.32 | 0% | 0.35 ± 0.48 | 1% |
|  | *CLOCKSCOR* | 4.55 ± 0.71 | 0% | 4.22 ± 1.0 | 1% |
|  | *COPYSCOR* | 4.82 ± 0.43 | 0% | 4.67 ± 0.65 | 0% |
|  | ***AVTOT6: RAVLT 6*** | 6.47 ± 3.83 | 1% | 3.65 ± 3.39 | 2% |
|  | **AVTOTB: RAVLT Interference** | 4.22 ± 1.77 | 1% | 5.00 ± 0.78 | 2% |
|  | ***Category Fluency*** | 18.43 ± 4.93 | 0% | 3.53 ± 1.69 | 0% |
|  | ***Boston Test Naming*** | 27.29 ± 2.81 | 0% | 25.12 ± 4.65 | 0% |
|  | ***AVDEL30: RAVLT delay*** | 5.04 ± 4.15 | 1% | 2.33 ± 3.19 | 2% |
|  | ***AVDELTOT: AVLT Recognition*** | 11.58 ± 2.99 | 0% | 9.54 ± 3.67 | 3% |
|  | ***Trail Making Test (Part A) – time*** | 36.81 ± 14.22 | 0% | 46.03 ± 18.47 | 0% |
|  | ***Trail Making Test (Part B) - time*** | 98.98 ± 51.82 | 1% | 136.86 ± 77.19 | 4% |
|  | *^(20)^****AVTOT15: RAVLT 15*** | 37.78 ± 10.44 | 1% | 29.84 ± 8.99 | 2% |
|  | *^(21)^Trail Making Test (Part B): Number of commission and omission errors* | 0.99 ± 2.29 | 1% | 1.72 ± 3.78 | 5% |
|  | *^(22)^Trail Making Test (Part A): Number of commission and omission errors* | 0.16 ± 0.45 | 0% | 46.03 ± 18.47 | 0% |
|  | ***^(23)^Forgetting Index*** | -23.41 ± 26.03 | 0% | -45.64 ± 36.39 | 2% |

*^(1)^ MMORIENTTEMP = MMDATE+MMYEAR+MMMONTH+MMDAY+MMSEASON ^(2)^MMORIENTSPAC=MMHOSPIT+MMFLOOR+MMCITY+MMAREA+ MMSTATE*

*^(3)^MMIMRECALL=MMBALL+MMFLAG+MMTREE*

*^4)^MMATTENTION=MMD+MML+MMR+MMO+MMW*

*^(5)^MMDLRECALL=MMBALLDL+MMFLAGDL+MMTREEDL*

*^(6)^MMLANGUAGE=MMWATCH+MMPENCIL*

*^(7)^MMLANGTOT=MMWATCH+MMPENCIL+MMREPEAT+MMHAND+MMFOLD+MMONFLR+MMREAD+MMWRITE+MMDRAW*

*^(8)^MMORIENTTOT=MMORIENTTEMP+MMORIENTSPAC*

*^(9)^MMCOMMAND=MMHAND+MMFOLD+MMONFLR*

*^(10)^MOCACLOCK=CLOCKCON+CLOCKNO+CLOCKHAN*

*^(11)^MOCANAMING=LION+RHINO+CAMEL*

*^(12)^MOCAMIMT1=IMMT1W1+IMMT1W2+IMMT1W3+IMMT1W4+IMMT1W5*

*^(13)^MOCAMIMT2=IMMT2W1+IMMT2W2+IMMT2W3+IMMT2W4+IMMT2W5*

*^(14)^MOCASERIAL=SERIAL1+SERIAL2+SERIAL3+SERIAL4+SERIAL5*

*^15)^MOCAREPEAT=REPEAT1+REPEAT2*

*^(16)^MOCAABS=ABSTRAN+ ABSMEAS*

*^(17)^MOCAMDL=DELW1+DELW2+DELW3+DELW4+DELW5*

*^(18)^MOCAORIETTEMP=DATE+MONTH+YEAR+DAY*

*^(19)^MOCAORIETSPAC=PLACE+CITY*

*^(20)^AVTOT15: RAVLT 15= AVTOT1+AVTOT2+AVTOT3+AVTOT4+AVTOT5*

*^(21)^Trail Making Test (Part B): Number of commission and omission errors= TRAAERRCOM+TRAAERROM*

*^(22)^Trail Making Test (Part A): Number of commission and omission errors=TRABERRCOM+TRABERROM*

*^(23)^ Forgetting Index = [(LM delayed recall – LM immediate)/LM immediate]*$\times$*100*
