# Supplementary material for: Neuropsychological predictors of conversion from mild cognitive impairment to Alzheimer’s disease: a feature selection ensemble combining stability and predictability
Source: BMC Med Inform Decis Mak. 2018 Dec 19;18:137. doi: 10.1186/s12911-018-0710-y (PMC6299964; doi:10.1186/s12911-018-0710-y)
Supplement: Supplementary file 2 — Individual and pairwise stability of the base FS algorithms used in the ensemble, using CCC data. (DOCX 61 kb) [file 12911_2018_710_MOESM2_ESM.docx]

**Table A.3.** Individual and pairwise stability of the base FS algorithms used in the ensemble. Results are averaged over the $10\times5$ stratified CV and $m$ subsets (for each possible subset size) for the 2-years (upper values), 3-years (middle values) and 4-years (bottom values), using CCC data.

|  | ReliefF | MIM | CMIM | MRMR | Chi-Squared | SVM-RFE | LL21 |
| --- | --- | --- | --- | --- | --- | --- | --- |
| ReliefF | 0.895±0.247  0.816±0.134  0.810± 0.139 | - | - | - | - | - | - |
| MIM | 0.608±0.343  0.521±0.230  0.505±0.273 | 0.913±0.232  0.859±0.096  0.859±0.132 | - | - | - | - | - |
| CMIM | 0.498±0.368  0.483±0.202  0.478±0.240 | 0.611±0.311  0.587±0.137  0.581±0.179 | 0.867±0.251  0.820±0.081  0.854± 0.161 | - | - | - | - |
| MRMR | 0.721±0.333  0.583±0.225  0.562±0.218 | 0.497±0.339  0.301±0.128  0.297±0.125 | 0.543±0.362  0.418±0.194  0.413±0.200 | 0.963±0.219  0.891±0.069  0.896±0.062 | - | - | - |
| Chi-Squared | 0.712±0.334  0.642±0.264  0.641±0.287 | 0.683±0.346  0.668±0.130  0.637± 0.207 | 0.399±0.399  0.514±0.159  0.415±0.243 | 0.564±0.331  0.289±0.193  0.402±0.175 | 0.954±0.225  0.915±0.084  0.916±0.171 | - | - |
| SVM-RFE | 0.339±0.349  0.217±0.096  0.202±0.096 | 0.309±0.357  0.158±0.106  0.136±0.107 | 0.210±0.376  0.135±0.066  0.098±0.050 | 0.368±0.340  0.230±0.085  0.219±0.086 | 0.211±0.119  0.184±0.07  0.187±0.135 | 0.365±0.341  0.371±0.100  0.327±0.087 | - |
| LL21 | 0.744±0.317  0.571±0.306  0.553±0.311 | 0.648±0.358  0.503± 0.274  0.495±0.291 | 0.451±0.364  0.389± 0.200  0.411±0.234 | 0.606±0.334  0.345±0.179  0.319±0.188 | 0.687±0.393  0.574±0.203  0.553±0.345 | 0.323±0.351  0.159±0.104  0.136±0.097 | 0.859±0.286  0.803±0.146  0.851±0.141 |
