# Supplementary material for: Neuropsychological predictors of conversion from mild cognitive impairment to Alzheimer’s disease: a feature selection ensemble combining stability and predictability
Source: BMC Med Inform Decis Mak. 2018 Dec 19;18:137. doi: 10.1186/s12911-018-0710-y (PMC6299964; doi:10.1186/s12911-018-0710-y)
Supplement: Supplementary file 3 — Stability and classification performance of classification models learnt with an incremental number of (ranked) features and using NB, DT, LR, SVM Poly and SVM RBF, per time windows, using ADNI and CCC data. RPT thresholds with β set as 0.1, 1 and 10 are illustrated. (DOCX 2920 kb) [file 12911_2018_710_MOESM3_ESM.docx]

**Figure A.1*.*** Stability and classification performance for multiple feature subset size (k) following $10\times5$ stratified CV and using time-windows of 2 years (upper), 3 years (middle) and 4 years (bottom) obtained with ADNI (left panel) and CCC (right panel) data, using NB. RPT thresholds with $\beta$ set as 0.1, 1 and 10 are illustrated.

**Figure A.2*.*** Stability and classification performance for multiple feature subset size (k) following $10\times5$ stratified CV and using time-windows of 2 years (upper), 3 years (middle) and 4 years (bottom) obtained with ADNI (left panel) and CCC (right panel) data, using DT. RPT thresholds with $\beta$ set as 0.1, 1 and 10 are illustrated.

**Figure A.3**. Stability and classification performance for multiple feature subset size (k) following $10\times5$ stratified CV and using time-windows of 2 years (upper), 3 years (middle) and 4 years (bottom) obtained with ADNI (left panel) and CCC (right panel) data, using LR. RPT thresholds with $\beta$ set as 0.1, 1 and 10 are illustrated.

**Figure A.4.** Stability and classification performance for multiple feature subset size (k) following $10\times5$ stratified CV and using time-windows of 2 years (upper), 3 years (middle) and 4 years (bottom) obtained with ADNI (left panel) and CCC (right panel) data, using SVMs with polynomial kernel of degree=2. RPT thresholds with $\beta$ set as 0.1, 1 and 10 are illustrated.

**Figure A.5.** Stability and classification performance for multiple feature subset size (k) following $10\times5$ stratified CV and using time-windows of 2 years (upper), 3 years (middle) and 4 years (bottom) obtained with ADNI (left panel) and CCC (right panel) data, using SVMs with RBF kernel. RPT thresholds with $\beta$ set as 0.1, 1 and 10 are illustrated.
